# Encapsulation of a Lactic Acid Bacteria Cell-Free Extract in Liposomes and Use in Cheddar Cheese Ripening

**DOI:** 10.3390/foods2010100

**Published:** 2013-03-13

**Authors:** Alice Beebyaanda Nongonierma, Magdalena Abrlova, Kieran Noel Kilcawley

**Affiliations:** 1 Teagasc, Food Research Centre, Moorepark, Fermoy, Co. Cork, Ireland; E-Mails: alice.nongonierma@ul.ie (A.B.N.); magdalena.abrlova@gmail.com (M.A.); 2 Department of Dairy and Fat Technology, Institute of Chemical Technology, Prague Technika5, Prague 6, 16628, Czech Republic

**Keywords:** Cheddar cheese, liposomes, cell-free extract, encapsulation, sensory

## Abstract

A concentrated form of cell free extract (CFE) derived from attenuated *Lactococcus lactis* supsb. *lactis* 303 CFE was encapsulated in liposomes prepared from two different proliposome preparations (Prolipo Duo and Prolipo S) using microfluidization. Entrapment efficiencies of 19.7 % (Prolipo S) and 14.0 % (Prolipo Duo) were achieved and the preparations mixed in the ratio 4 (Prolipo Duo):1 (Prolipo S). Cheddar cheese trials were undertaken evaluating the performance of CFE entrapped in liposomes, empty liposomes and free CFE in comparison to a control cheese without any CFE or liposomes. Identical volumes of liposome and amounts of CFE were used in triplicate trials. The inclusion of liposomes did not adversely impact on cheese composition water activity, or microbiology. Entrapment of CFE in liposomes reduced loss of CFE to the whey. No significant differences were evident in proteolysis or expressed PepX activity during ripening in comparison to the cheeses containing free CFE, empty liposomes or the control, as the liposomes did not degrade during ripening. This result highlights the potential of liposomes to minimize losses of encapsulated enzymes into the whey during cheese production but also highlights the need to optimize the hydrophobicity, zeta potential, size and composition of the liposomes to maximize their use as vectors for enzyme addition in cheese to augment ripening.

## 1. Introduction

Acceleration of cheese ripening has been proposed as a way to produce a fast ripening curd for processed cheese or to reduce costs associated with cheese manufacture [1]. Different strategies for acceleration of cheese ripening have been described with the addition of exogenous enzymes being the most studied technique [1,2,3]. The addition of exogenous enzymes is an accepted method to accelerate Cheddar cheese ripening, however significant amounts of enzyme are lost to the whey and can have adverse effects on whey quality [1]. Hence, the use of encapsulated enzymes has evolved as a method to combat losses to whey [4] and improve enzyme retention in the curd [5]. Previous studies have identified liposomes as suitable vectors for the inclusion of enzymes into cheese as they have a high affinity for milk fat and can encapsulate sufficiently large quantities of water soluble material [6].

Previous studies have described the acceleration of cheese ripening with enzymes encapsulated in liposomes [7,8,9,10,11,12,13]. Food-grade water soluble enzymes can be encapsulated in liposomes using milk fats or different food-grade proliposomes [6,14], and losses to whey may be minimized as liposomes partition with the fat globules and the casein matrix when added to the milk [1]. Microfluidization, is a homogenization method based on the use of relatively high pressures, and has been described in the manufacture of liposomes [7,14,15,16]. In contrast with other liposome preparation methods, microfluidization does not require organic solvents and is easy to scale up, making it suitable for large scale food grade applications [7,15,17].

The objectives of this study were to evaluate the impact of the addition of cell-free extracts (CFE) of *Lactococcus lactis* subsp. *lactis* 303 and liposome-encapsulated CFE of *Lactococcus lactis* subsp. *lactis* 303 on the ripening of Cheddar cheese. CFE of *Lactococcus lactis* subsp. *lactis* 303 was produced by microfluidization and subsequently encapsulated in two liposome preparations with different phospholipid compositions. Losses of key intracellular enzymes were monitored during production and ripening together with, microbiological analyses and physico-chemical characteristics of the cheeses (composition, water activity and microscopy). Descriptive sensory analysis and volatile profiling were also determined at 112 days ripening.

## 2. Results and Discussion

### 2.1. Lactococcus lactis subsp. lactis 303 CFE Manufacture and Encapsulation in Liposomes

Total cell counts in 10% reconstituted skim milk (RSM) before tyndallization were 1.0 × 10^3 ^CFU/mL after which bacteria were not detected (data not shown). *Lactococcus lactis* subsp. *lactis* 303 cells were grown to 1.1 × 10^10^ CFU/mL (Table 1) and following microfiltration (MF) the retentate was processed through the microfluidizer and subsequently freeze-dried. There was a small decrease in cell numbers post microfluidization (9.0 × 10^9^ CFU/mL) (Table 1).

**Table 1:** Total cell counts and enzyme activities of post-proline dipeptidyl aminopeptidase (PepX) and lactate dehydrogenase (LDH) determined at the different stages of the cell free extract (CFE) preparation.

Sample	Total cell counts (CFU/mL or CFE/g) *	PepX (mM AMC/min·mL or CFE/g) *	LDH (units/mL or CFE/g) *
In RSM after inoculation	5.0 × 10^6 b^	nd	nd
In RSM after growth	1.1 × 10^10 c^	3.77 ± 0.01 ^a^	0.00 ± 0.00 ^a^
After microfluidization (4000 psi)	9.0 × 10^9 c^	25.20 ± 0.01 ^b^	1.31 ± 0.14 ^b^
Freeze-dried microfluidized cells **	4.2 × 10^10 c^	208.69 ± 14.75 ^c^	13.8 ± 0.37 ^c^
Freeze-dried CFE **	0 ^a^	1333.1 ± 116.07 ^d^	nd

* Within each column, values with different superscript letters are significantly different (*p* < 0.05); ** Values for freeze-dried samples are expressed per g of freeze-dried powder, all other values are expressed per mL; nd: Not determined; RSM: Reconstituted skim milk; CFU: Colony-forming units; AMC: 7-Amino-4-methyl coumarin.

In the RSM after growth, low level of PepX activity was determined but no LDH activity was detected indicating minimal lysis of the bacterial cells at this stage (Table 1). After one pass through the microfluidizer, there was a significant increase in both PepX and LDH activities (*p* < 0.05) and ~18% reduction of viable cells due to cell disruption of *Lactococcus lactis* subsp. *lactis* 303 cells. The total cell counts, PepX and LDH activity increased in the microfluidized cells after freeze drying due to a concentration effect and additional cell lysis as cell counts and enzyme activities are expressed on weight basis rather than on a volume basis. No viable cells were present in the freeze dried CFE as anticipated and the level of Pep X activity was very high due to the fact that it was concentrated in the cell extract (Table 1). This result also highlights the fact that it is possible to freeze dry CFE and maintain peptidase activity.

Encapsulation of *Lactococcus lactis* subsp. *lactis* 303 CFE was undertaken in two types of proliposomes (Duo and S). The amount of PepX activity in the water soluble 303 CFE used to manufacture the Cheddar cheese samples is given in Table 2. The Prolipo Duo preparation has a lower zeta potential than Prolipo S (Table 2) preparation because it contains more negatively charged phospholipids. Lower zeta potential values are associated with greater liposomal stability. A liposome with a low zeta potential can cause electrostatic repulsions, which in turn may prevent destabilization processes, such as coalescence and aggregation [6]. During the encapsulation process, a substantial amount of CFE remained unbound to the liposomes (>70%). The encapsulation efficiency of 303 CFE was 19.7% for Prolipo S and 14.0% for Prolipo Duo (Table 2) which was not significantly different (*p* ≥ 0.05). Encapsulation efficiency of CFE in liposomes made with Prolipo S up to 58.4% has been reported for a cell free extract of *Lactobacillus casei* subsp. *pseudoplantarum* [12] and lower encapsulation efficiency in liposomes of 12.7% for cryopsin has been described [13]. Nongonierma *et al*. [14] reported enzyme entrapment efficiencies of 62.7% in Prolipo C and 29.2% in Prolipo S for Debitrase DBP20. In Prolipo VPF 012, encapsulation efficiencies of 32%–36% have been reported for bacterial and fungal proteinases [8], 35.9% for Palatase M and 40.3% for Lipase 50 [10]. Liposomal encapsulation as low as 12.7% for cryopsin was shown to accelerate proteolysis in Manchego cheese [13], therefore the encapsulation efficiencies achieved in this study were deemed appropriate to positively influence acceleration of proteolysis and thus flavor in Cheddar cheese.

**Table 2:** Zeta potential and enzyme activity of post-proline dipeptidyl aminopeptidase (PepX) from *Lactococcus lactis* ssp. *lactis* 303 cell-free extract (CFE) encapsulated in the liposomes S and Duo at the levels which were used for the cheese trials. Each value is the average of triplicate determinations (*n* = 3).

	Zeta potential	Total activity		Unbound		Encapsulated		
		PepX activity *	% total activity	PepX activity *	% total activity	PepX activity *	% total activity	
Prolipo S	−17.0	822.1 ± 273.1	100.0	610.4 ± 216.1	74.2 ^a^	162.2 ± 33.4	19.7 ^b^	
Prolipo Duo	−39.3	3275.0 ± 1073.8	100.0	2448.4 ± 905.6	74.8 ^a^	459.4 ± 144.5	14.0 ^b^	

* PepX activity is expressed in µmol pNA/min·mL; Values with different superscript letters are significantly different (*p* < 0.05).

The final preparation used in the cheese-making trials contained a mixture of both Proliposome preparations at a ratio of 4 parts Prolipo Duo and 1 part Prolipo S. This mixture was utilized as the Prolipo Duo liposomes are more hydrophobic than the Prolipo S and should partition better with the milk fat globule membrane and the casein matrix during cheese production increasing retention in the cheese curd [1,6]. It has been suggested that liposomes are distributed in the curd in the same fashion as bacterial cells [3], where liposomes behave as a carrier for different enzyme activities similarly to bacterial cells. However, it is believed that enzyme release from liposomes occurs at a faster rate than from bacterial cells [3,18]. Although the mechanisms of enzyme release from liposomes in cheese are poorly understood [5]. It has been suggested that enzyme release from liposomes in cheese may involve various parameters including temperature, pH and ionic strength [9]. In addition, it is thought that liposome degradation in cheese may occur following aggregation processes which are favored at low pH values. It has been shown that a decrease from pH 7 to 5 was responsible for the release of active agent (calcein) encapsulated in liposomes [19]. Several studies have demonstrated the potential of liposome encapsulated enzymes as a means to accelerate cheese ripening [10,11,12], by reducing losses to whey [4].

### 2.2. Influence of CFE and Encapsulated CFE on the Composition and Water Activity of the Cheeses

The composition of the cheeses was determined at day 14 (Table 3). No significant differences (*p* ≥ 0.05) were evident for moisture, fat, protein, salt or pH between the four cheeses (Table 3). This suggested that addition of encapsulated or non-encapsulated 303 CFE did not adversely impact on the composition of the cheese, which is in agreement with Bainville *et al*. [20].

**Table 3:** Composition of the cheese samples determined at day 14 of ripening. Each value is the average of triplicate determinations (*n* = 3). Cheese 1, Control; Cheese 2, cheese with empty liposomes S and Duo; Cheese 3, cheese with liposomes S and Duo containing the encapsulated *Lactococcus lactis* ssp. *lactis* 303 cell-free extract (CFE); Cheese 4, cheese with *Lactococcus lactis* ssp. *lactis* 303 CFE.

	Moisture (% w/w)	Fat (% w/w)	Protein (% w/w)	Salt (% w/w)	Water activity	pH
Cheese 1	39.19 ± 1.03 ^a^	29.63 ± 0.68^ a^	24.95 ± 0.62^ a^	1.79 ± 0.23^ a^	0.967 ± 0.002^ a^	5.12 ± 0.11^ a^
Cheese 2	39.29 ± 0.96^ a^	29.78 ± 0.65^ a^	24.87 ± 0.44^ a^	1.72 ± 0.17^ a^	0.972 ± 0.005 ^a^	5.08 ± 0.07^ a^
Cheese 3	39.31 ± 0.83^ a^	29.67 ± 0.68^ a^	24.82 ± 0.32^ a^	1.74 ± 0.12^ a^	0.969 ± 0.002 ^a^	5.12 ± 0.09^ a^
Cheese 4	38.68 ± 0.89^ a^	29.92 ± 0.58^ a^	25.19 ± 0.61^ a^	1.95 ± 0.22^ a^	0.970± 0.002 ^a^	5.16 ± 0.12^ a^

Within the same column, values with similar superscript letters are not significantly different (*p* ≥ 0.05).

Some textural defects have also been associated with addition of liposomes into cheeses, which is thought to be due to an increase in cheese moisture [7,8,10,11,12]. An increase in the cheese moisture has been associated with water binding at the liposome surface [12]. In addition to the increased moisture, the associated decrease in the protein content can lead to a less firm and more brittle cheese structure [8]. In contrast, Lariviere *et al*. [7] showed that apart from increased moisture levels, cheeses with liposomes did not have any negative textural issues in comparison to a control cheese without liposome addition. No significant differences (*p* ≥ 0.05) were noted in water activity between the cheeses, highlighting that not only was there no differences in moisture, but that water activity was not altered by the inclusion of liposomes. Lower water activity is known to reduce rates of proteolysis in cheese [21].

### 2.3. Enumeration of Starter and Non-Starter Lactic Acid Bacteria and Enzyme Activities during Cheese Ripening

**Figure 1:**
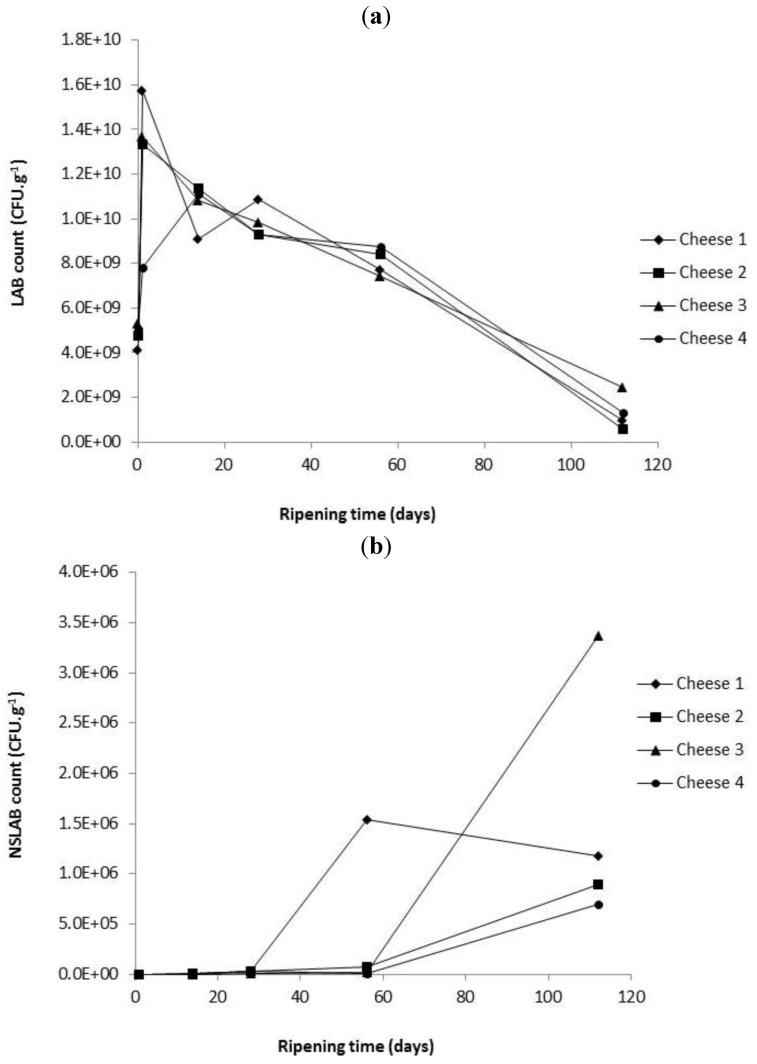
Microbiological count in different cheeses for (**a**) lactic acid bacteria (LAB) and (**b**) non-starter lactic acid bacteria (NSLAB) as a function of ripening time. Each point is the average of three determinations (*n* = 3). Cheese 1, Control; Cheese 2, cheese with empty liposomes S and Duo; Cheese 3, cheese with liposomes S and Duo containing the encapsulated *Lactococcus lactis* ssp. *lactis* 303 cell-free extract (CFE); Cheese 4, cheese with *Lactococcus lactis* ssp. *lactis* 303 CFE.

Evolution of LAB and NSLAB were monitored during the ripening in each cheese (Figure 1). As anticipated, LAB counts (Figure 1a) decreased and NSLAB increased during ripening (Figure 1b). The decrease in LAB has been attributed to the changes in the cheese matrix including reduction in pH, lactose content and an increase in salt concentration [5]. There were no significant differences in the cell counts determined for the four cheeses both for LAB and NSLAB (*p* ≥ 0.05).

**Figure 2:**
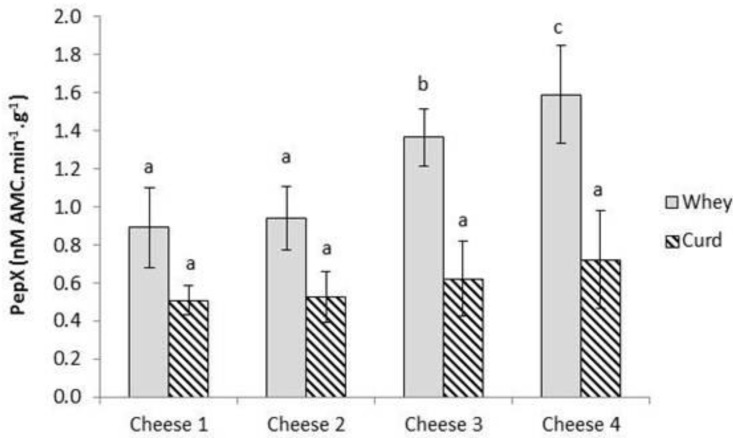
Post-proline dipeptidyl aminopeptidase (PepX) in the curd and whey from the different cheese samples. Each point is the average of three PepX determinations (*n* = 3). For the whey or curd samples, figures with different letters are significantly different (*p* < 0.05). Cheese 1, Control; Cheese 2, cheese with empty liposomes S and Duo; Cheese 3, cheese with liposomes S and Duo containing the encapsulated *Lactococcus lactis* ssp. *lactis* 303 cell-free extract (CFE); Cheese 4, cheese with *Lactococcus lactis* ssp. *lactis* 303 CFE.

PepX and LDH activities were measured in the curd and whey samples during production of each cheese. Only residual activities for LDH from starter LAB were found as anticipated (data not reported), as cell lysis does not normally occur in the early stages of cheese making [22]. There were no significant differences between PepX activities of the different curd samples (*p* ≥ 0.05), but there were significant differences (*p* < 0.05) in PepX activity in the whey samples (Figure 2).

Significantly higher PepX activities were measured in the whey in Cheeses 3 and 4, compared to Cheeses 1 and 2 (*p* ≥ 0.05). As Cheeses 1 and 2 did not contain additional CFE it was anticipated that levels should be similar and lower than Cheeses 3 and 4. The fact that levels of Pep X are significantly higher (*p* < 0.05) in Cheese 4 highlights that significant amounts of the added free CFE were lost to the whey at drainage. The fact that additional levels of PepX activity were not found in curds and that lower levels were in the whey in Cheese 3 in comparison to Cheese 4 indicates that additional CFE is incorporated into the curd within the liposome preparations.

**Figure 3:**
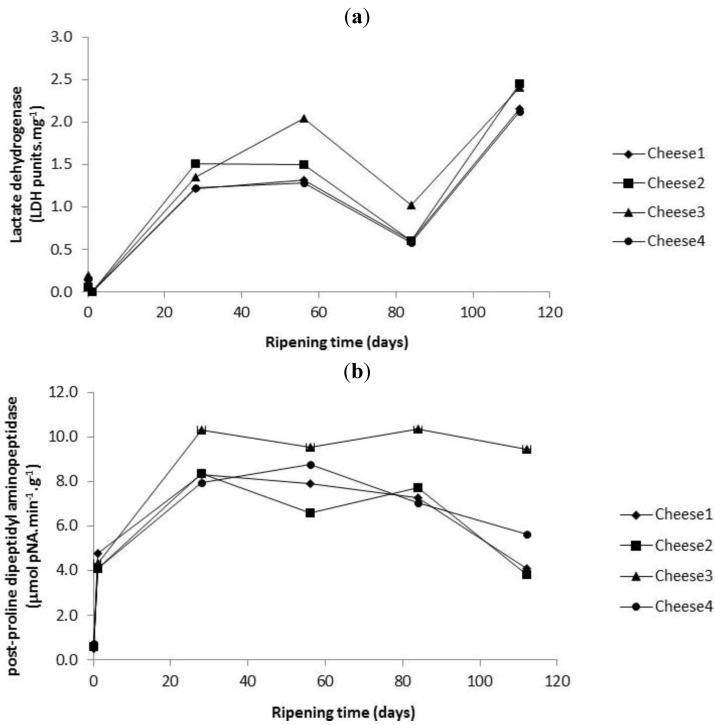
(**a**) Lactate dehydrogenase (LDH) activity in the different cheese samples as a function of ripening time. (**b**) Post-proline dipeptidyl aminopeptidase (PepX) activity in the different cheese samples as a function of ripening time. Each point is the average of three determinations (*n* = 3). Cheese 1, Control; Cheese 2, cheese with empty liposomes S and Duo; Cheese 3, cheese with liposomes S and Duo containing the encapsulated *Lactococcus lactis* ssp. *lactis* 303 cell-free extract (CFE); Cheese 4, cheese with *Lactococcus lactis* ssp. *lactis* 303 CFE.

PepX and LDH activities were monitored in the different cheeses over 112 days of ripening. There were no significant (*p* ≥ 0.05) differences between the four cheese for PepX or LDH activity. Both LDH and PepX (Figure 3a,b) activities increased numerically during ripening. LDH levels increased up to day 56 and then dropped up to day 84 and increased again up to day 112 in all cheeses (Figure 3a). The initial increase in LDH activity is likely related to lysis of starter LAB (Figure 1a), and the later increase presumably due to a combination of continued lysis of starter LAB and lysis of NSLAB. Even though NSLAB were seen to numerically accumulate (Figure 1b) at this time point, it is anticipated that a percentage will also autolyse [23]. PepX activity increased rapidly between day 0 and 28 and then leveled off (Figure 3b).

### 2.4. Evolution of Proteolysis in Cheeses over Ripening

**Figure 4:**
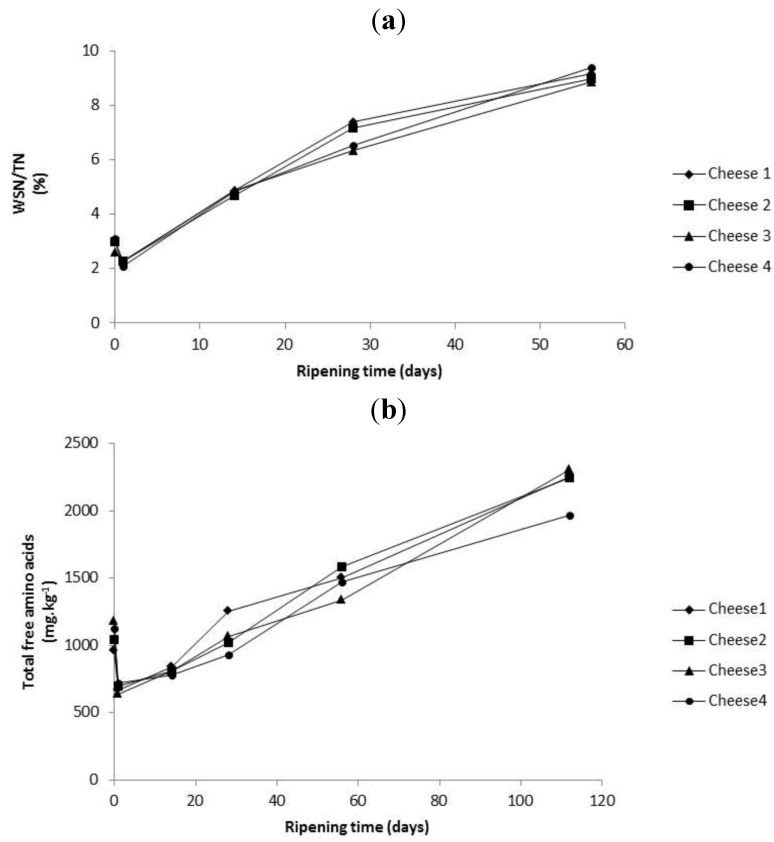
(**a**) pH 4.6 Water soluble nitrogen/total nitrogen (WSN/TN) as a function of ripening time of the different cheeses. (**b**) Average total free amino acids (TFAA). Each point is the average of three determinations (*n* = 3). Cheese 1, Control; Cheese 2, cheese with empty liposomes S and Duo; Cheese 3, cheese with liposomes S and Duo containing the encapsulated *Lactococcus lactis* ssp. *lactis* 303 cell-free extract (CFE); Cheese 4, cheese with *Lactococcus lactis* ssp. *lactis* 303 CFE.

No significant differences (*p* ≥ 0.05) could be determined for pH 4.6 water soluble nitrogen/total nitrogen (WSN/TN%) (Figure 4a), or total free amino acids (TFAA) (Figure 4b), between all four cheeses during ripening. No difference in primary proteolysis as measured by pH 4.6 WSN/TN% was anticipated, as the CFE added in Cheeses 3 and 4 contained only peptidase activity and as the added liposomes in Cheeses 2 and 3 did not influence composition or water activity, levels of primary proteolysis should not be different between these cheeses. However, it was anticipated that levels of secondary proteolysis would have been significantly higher in Cheese 3 and maybe Cheese 4 due to additional levels of added CFE in encapsulated or in free form, respectively. As no TFAA differences were evident and no statistical differences were found in PepX activity between the cheeses this indicates that the concentration of CFE added in Cheeses 3 and 4 was either (1) insufficient to influence secondary proteolysis, (2) most PepX activity was lost at whey drainage (Figure 2), a definite factor in Cheese 4, or (3) in the case of Cheese 3, the liposomes remained intact throughout ripening and did not release sufficient additional Pep X activity (Figure 3b). It is notable that as differences of PepX lost to the whey were significantly less in Cheese 3, and levels within the curd in Cheese 3 and 4 were similar, this suggests that the liposomes in Cheese 3 did not rupture significantly during ripening.

### 2.5. Cryo SEM of Liposomes within Cheese Curd up to 28 Days of Ripening

**Figure 5:**
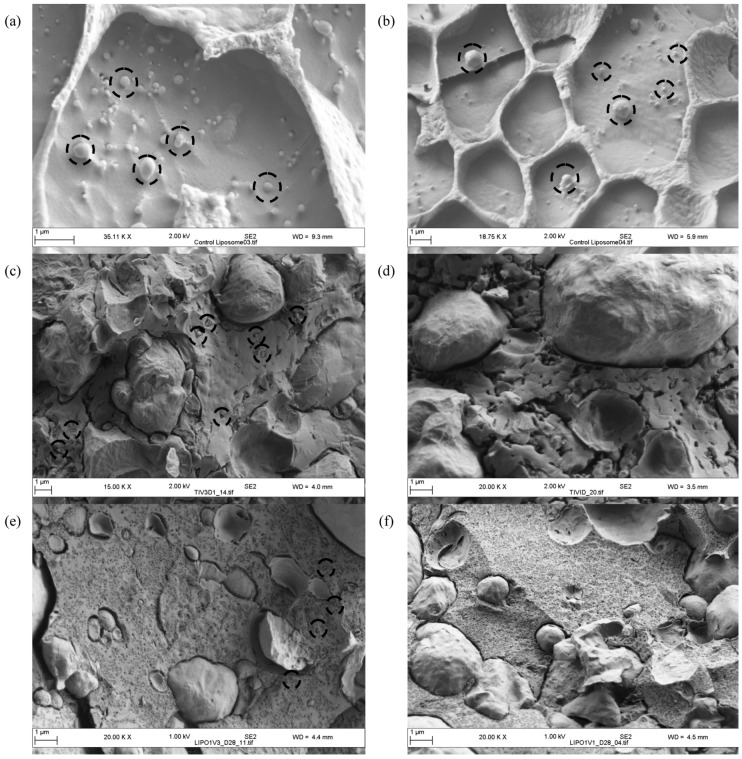
(**a**) and (**b**), cryogenic-scanning electron micrography (Cryo SEM) of liposomes. Cryo SEM of the control cheese (Cheese 1) and the cheese with liposomes S and Duo containing the encapsulated *Lactococcus lactis* ssp. *lactis* 303 cell-free extract (Cheese 3). Cryo SEM at day 1 for (**c**) Cheese 3 and (**d**) Cheese 1 and at day 28 for (**e**) Cheese 3 and (**f**) Cheese 1. Some liposomes are circled on micrographs.

Cryo SEM was utilized to visualize the liposomes after manufacture and to determine their presence and location in the cheese. The liposomes had a heterogonous size as seen in the micrographs on Figure 5a,b. Similar results have already been reported with liposomes manufactured with Prolipo S and C showing a bimodal distribution made of populations of small (30–40 nm) and large vesicles (300–700 nm) [14].

Analysis of cheeses with (Cheese 3) and without (Cheese 1) liposomes was carried out to visualize the liposomes in the cheese at day 1 and 28. Granular particles seen in Cheese 3 (Figure 5c) and absent from Cheese 1 (Figure 5d) at day 1 were identified as liposomes. At day 28, liposomes could be clearly seen on the micrograph of Cheese 3 (Figure 5e) which again were absent in Cheese 1 (Figure 5f), as expected. The micrographs confirmed that liposomes partitioned with the curd during cheese production. The liposomes could still be visualized as intact particles within the cheese mass after 28 days ripening, we did not analyze any cheese samples later in ripening as we were anticipating early rupture based on previous studies [7,8]. In agreement with previous findings, the liposomes seen on the micrographs were located at the fat-casein interface and also existed as single particles which were distributed throughout the cheese curd, but were fully intact at day 28.

### 2.6. Descriptive Sensory Analysis and Volatile Profiles of Cheeses at Day 112

The sensory attributes and volatile profiles of each cheese were studied at day 112. The relative concentrations of identified aroma compounds in each cheese are reported in Table 4.

**Table 4:** Concentration of volatile compound in the different Cheddar cheeses relative to the internal standard 2-methyl-3-heptanone. Cheese 1, Control; Cheese 2, cheese with empty liposomes S and Duo; Cheese 3, cheese with liposomes S and Duo containing the encapsulated *Lactococcus lactis* ssp. *lactis* 303 cell-free extract (CFE); Cheese 4, cheese with *Lactococcus lactis* ssp. *lactis* 303 CFE.

Volatiles	Concentration of volatiles (µg/kg) in the different cheese samples *			
	Cheese 1	Cheese 2	Cheese 3	Cheese 4
Methanethiol	2.18 ^a^	3.80 ^a^	3.86 ^a^	3.02 ^a^
Ethyl alcohol	196.78 ^b^	347.96 ^a, b^	350.35 ^a^	248.24 ^a, b^
Propanol	355.71 ^a, b^	373.39 ^a, b^	55.58 ^b^	686.59 ^a^
Carbon disulfide	24.73 ^a^	15.69 ^a^	21.22 ^a^	17.24 ^a^
2-Butanone	641.36 ^a^	654.36 ^a^	400.35 ^a^	977.05 ^a^
2-Butanol, (*R*)-	736.40 ^a^	776.54 ^a^	1597.63 ^a^	1096.35 ^a^
2-Pentanone	13.95 ^b^	28.24 ^b^	86.34 ^a^	33.04 ^b^
Acetoin	39.43 ^a^	148.11 ^a^	134.13 ^a^	54.22 ^a^
Methyl butanoate	5.60 ^a^	7.17 ^a^	9.02 ^a^	6.26 ^a^
Ethyl butanoate	42.24 ^b^	374.76 ^a^	145.41 ^a, b^	184.72 ^a, b^
Pentanoic acid	3.94 ^a^	6.17 ^a^	7.47 ^a^	5.20 ^a^
2-Heptanone	17.63 ^b^	38.24 ^a^	25.44 ^a, b^	26.69 ^a, b^
Heptanal	1.87 ^b^	4.32 ^a^	2.88 ^a, b^	2.75 ^a, b^
2,6-Dimethyl-pyrazine	0.00 ^b^	0.00 ^b^	0.00 ^b^	0.23 ^a^
Methyl hexanoate	5.47 ^a^	7.95 ^a^	8.46 ^a^	5.85 ^a^
Dimethyl trisulfide	10.25 ^a^	20.27 ^a^	14.98 ^a^	8.90 ^a^
Ethyl hexanoate	7.13 ^a^	11.79 ^a^	11.22 ^a^	9.38 ^a^
Benzeneacetaldehyde	2.50 ^a^	5.13 ^a^	5.05 ^a^	2.54 ^a^
2-Nonanone	2.95 ^b^	6.63 ^a^	3.99 ^a, b^	4.84 ^a, b^
Nonanal	5.73 ^a^	16.30 ^a^	4.04 ^a^	8.73 ^a^
2-Ethyl-hexanoic acid	0.73 ^a^	1.07 ^a^	1.42 ^a^	0.68 ^a^
Methyl octanoate	0.96 ^a^	1.11 ^a^	0.93 ^a^	0.70 ^a^
Hexanoic acid	10.17 ^a^	14.85 ^a^	9.16 ^a^	7.48 ^a^
2,5-Dihydro-3-methyl-furan	4.35 ^a^	5.45 ^a^	4.20 ^a^	2.87 ^a^
Ethyl octanoate	0.65 ^a^	1.26 ^a^	1.18 ^a^	1.15 ^a^

* Within the same row, compound concentration with a different superscript letter are significantly different (*p* < 0.05).

**Figure 6:**
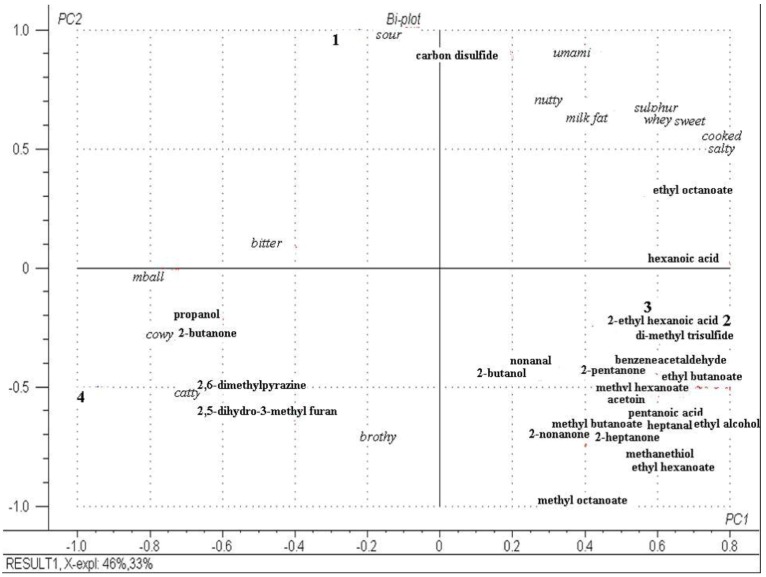
Principal component plot of cheese volatile compounds and of descriptive analysis of cheese flavors (PC1 and PC2) evaluated at day 112. Numbers refer to cheeses: 1, Control; 2, cheese with empty liposomes S and Duo; 3, cheese with liposomes S and Duo containing the encapsulated *Lactococcus lactis* ssp. *lactis* 303 cell-free extract (CFE); 4, cheese with *Lactococcus lactis* ssp. *lactis* 303 CFE.

The different volatile compounds originate from free fatty acids, carbohydrates and amino acids [24,25]. Similar volatiles have previously been identified in Cheddar cheese [26]. Many volatile compounds were found at the same concentration in all cheeses, but some significant (*p* < 0.05) differences were apparent. Significant differences (*p* < 0.05) in sensory attributes were also apparent between the cheeses at day 112. Figure 6 is a PCA biplot that best discriminates each cheese at day 112 based on both their sensory attributes and volatile compounds. Cheeses 2 and 3 were closely associated with each other, but not with any particular sensory attributes. However, they were associated with the most volatiles compounds; acid (2-ethyl hexanoic acid), esters (methyl butanoate and methyl hexanoate), ketones (2-pentanone and acetoin), aldehydes (nonanal and benzeneacetaldehyde), alcohol (2-butanol) and sulphur (di-methyl tri-sulphide) compounds. Cheese 4 was associated with the sensory attributes “cowy”, “mothball” and “catty”, some of which are associated with more aged Cheddar cheese [27,28] and the volatiles; alcohol (propanol), ketone (2-butanone), pyrazine (2,6-di-methyl pyrazine) and furan (2,5-di-hydro-3-methyl furan). Cheese 1 was associated with the sensory attribute “sour” and the sulphur compound Carbon disulphide and clearly had the least developed flavor.

## 3. Experimental Section

### 3.1. Preparation of Lactococcus lactis subsp. lactis 303 CFE

A volume of 15 L of 10 % (w/v) reconstituted skimmed milk (RSM-Kerry Ingredients, Co. Cork, Ireland) was prepared by dispersing skimmed milk powder in deionized water at room temperature. This was transferred to a sterile 18 L fermentor (Braun Biostat ED, Melsungen, Germany). The milk was sterilized using a tyndalization procedure where the 10% RSM was heated 3 times at 100 °C for 15 min and cooled down to 23 °C, over 3 days. This heat treatment did not cause any visible change in the media. Samples were analyzed for total cell counts 24 h after heat treatment. The 10% RSM was aseptically inoculated with *Lactococcus lactis* subsp. *lactis* 303 strain at 0.1%, v/v (*i.e.*, 15 mL of *Lactococcus lactis* subsp. *lactis* 303 culture in the 15 L RSM). The *Lactococcus lactis* subsp. *lactis* 303 cells were grown in 10% RSM until the pH reached 5.2 (~17 h) without agitation at 23 °C. The grown culture was agitated at 100 rpm and an aqueous solution of NaOH 1 M (Fisher Scientific Ireland, Dublin, Ireland) was aseptically added until pH 7.0 was achieved. The culture was concentrated by microfiltration (Techsep PS3R, Novasep, Pompey, France) using a ceramic membrane M14 (0.14 µm pore diameter). The inlet and outlet pressures of the microfiltration unit were set at 2.5 and 2.0 bars, respectively. Microfiltration was carried out for ~6 h with a final retentate volume of ~3.5 L and a permeate volume of ~5.5 L. The concentrated culture was microfluidized using a Microfluidics M-110-EH-30 (Microfluidics, Newton, MA, USA) equipped with a Y interaction chamber at 4000 psi at 16 °C under aseptic conditions. The microfluidized mixture was freeze-dried using a Labconco Freezone 12 Plus dryer (Fisher Scientific). The freeze dried powder was vacuum packed sealed and stored at −20 °C until required.

### 3.2. Preparation of Lactococcus lactis subsp. lactis 303 CFE

A water soluble extract of *Lactococcus lactis* subsp. *lactis* 303 (303) cells was prepared by suspending the freeze-dried 303 in 25 mM Tris-HCl buffer at pH 7.4 to a final concentration of 10% (w/w) at room temperature. The insoluble part of the enzyme preparation was removed by centrifugation using a Sorvall 5U (Unitech, Dublin, Ireland) at 3500× *g*, 15 min at 21 °C and the supernatant was recovered.

### 3.3. Encapsulation of CFE in Liposomes

Two different food grade Proliposome preparations were studied: Prolipo S and Prolipo Duo (Lucas Meyer Cosmetics, Champlan, France). Prolipo S contains 30% (w/w) of unsaturated soybean phospholipids and 70% (w/w) of aqueous media (a mixture of ethanol or water and glycerol). Prolipo Duo contains 50% (w/w) of unsaturated soybean phospholipids and 50% (w/w) of aqueous media [14,29]. The liposomes were prepared according to the procedure of Dufour *et al*. [9] and as described in Nongonierma *et al*. [14]. Briefly, 64 g of Prolipo Duo or 16 g of Prolipo S were mixed with 256 mL or 64 mL, respectively of the 10% (w/v) 303 CFE solution under agitation with an overhead stirrer RW 20DZM (Janke and Kunkel, Staufen, Germany) at 300 rpm for 15 min. A volume of 480 mL and 120 mL of 25 mM Tris-HCl at pH 7.4 was added to the Prolipo Duo/CFE and the Prolipo S/CFE preparations, respectively and agitated at 500 rpm for 15 min using the overhead stirrer. Each preparation was processed in one pass through the M-110-EH-30 Microfluidizer (Microfluidics) equipped with a Y interaction chamber at 4000 psi, at 16 °C. Liposomes without CFE were prepared in the same fashion as described above using an equivalent volume of 25 mM Tris-HCl buffer at pH 7.4 in place of the CFE. The zeta potential of the liposomes was measured at 21 °C, with photon correlation spectrometry using a Zetasizer Nano ZS (Malvern Instruments, Worcestershire, UK) as per Nongonierma *et al*. [14].

### 3.4. Cheese Manufacture

The starter cultures used for the production of all cheeses was *Lactococcus lactis* ssp. lactis 303 (Chr. Hansen Ireland Limited, Little Island, Ireland). The culture was maintained in 10% RSM at −80 °C and grown overnight at 23 °C in heat-treated RSM until the pH reached ~4.9. The inoculum level was 1.5% (v/v) of *Lactococcus lactis* ssp. lactis 303 per 250 L of cheesemilk.

The liposome preparations, were mixed in the following ratio; 4 (Prolipo Duo):1 (Prolipo S) (Lipo) and 1 L was added to 250 L of the milk immediately after the addition of the starter. An experimental design was used to study the impact of the addition of 303 CFE and encapsulated 303 CFE on cheese ripening. The experimental design was as follows; Cheese 1 did not have any 303 CFE or Lipo added; Cheese 2 contained only Lipo, Cheese 3 contained Lipo plus encapsulating 303 CFE and Cheese 4 contained only 303 CFE. The volume of added material was identical in all cases. Cheddar cheeses were manufactured in triplicate using a standard cheese making procedure described by Wilkinson *et al*. [30]. The rennet ChyMax Plus (Chr. Hansen) was used at a rate of 18 mL per 100 L of milk. Cheddar cheeses were produced in triplicate at pilot scale and the vats were rotated for each cheese trial to avoid contribution from any vat-related factors. On the day of production, bulk whey and bulk curd were sampled for microbiology, enzymology and proteolysis. Cheddar cheeses were packed under vacuum at day 1 and ripened at 8 °C for 112 days. The cheeses were sampled at day 1, 14, 28, 56 and 112 for determination of enzyme activities and microbiological analyses. Composition of the cheese was assessed at day 14.

### 3.5. Determination of the Enzyme Activity

#### 3.5.1. Determination of Enzyme Activities in the CFE Samples

For the samples generated during the manufacture of the CFE, two enzyme assays were carried out: post-prolyl di-peptidyl aminopeptidase (PepX) and lactate dehydrogenase (LDH). The enzyme activity was measured in the inoculated RSM before and after *Lactococcus lactis* subsp. *lactis* 303 growth, in the microfiltration permeate and retentate and in the different microfluidized samples. PepX activity was measured in curd or whey samples based on their ability to hydrolyse Gly-Pro-7-amino-4-methyl coumarin (AMC) (Bachem, Bubendorf, Switzerland). The assay was carried out as modifications of the methods described by Habibi-Najafi and Lee [31] and Kilcawley *et al*. [32]. The substrate was made up to 0.111 mM in 50 mM Tris-HCl buffer pH 7.0. A standard curve was prepared 0–1000 nM AMC. The reaction mixture consisted of 50 μL of microfluidized sample which was added with 450 μL of substrate and 500 μL of 50 mM Tris-HCl buffer pH 7.0. The reaction was carried out at 37 °C for 15 min. The release of AMC was monitored by fluorometry (Varian Cary Eclipse Fluorometer, JVA Analytical Ltd., Dublin, Ireland) at the excitation 370 nm and emission 440 nm. Each sample was assayed in triplicate, with a blank consisting of 50 µL of 50 mM Tris-HCl buffer pH 7.0. The reaction was terminated by the addition of 250 µL of 1.5 M acetic acid (Sigma Aldrich, Dublin, Ireland). The fluorescence reading of the blank was subtracted from that of the samples. The release of AMC was calculated by reference to the standard curve where the fluorescence of the sample was converted to nmol of AMC. PepX activity was expressed as µmol/min·mL of sample.

LDH activity was measured as per Cogan *et al*. [33] by following the rate of change in absorbance at 340 nm due to the enzymatic oxidation of pyruvate in the presence of nicotinamide adenine dinucleotide (NADH, Sigma Aldrich). LDH activity was determined by mixing 2.7 mL of Tris-maleate buffer, pH 7.0 (Sigma Aldrich) with 0.1 mL of 4.5 mM NADH, 0.1 mL of 30 mM Fructose 1,6 bis-phosphate (Sigma Aldrich) and 0.1 mL of sample. The reaction was started by the addition of 0.1 mL of 300 mM pyruvate (Sigma Aldrich) and the absorbance at 340 nm (Varian Cary Bio-100 UV/Vis Spectrometer, JVA Analytical Ltd.) was monitored for 90 s. One unit of LDH was defined as the amount of enzyme required to catalyze the oxidation of 1 µM of NADH/min·mL of sample.

#### 3.5.2. Determination of PepX Activity in the Liposomes

For the liposomes, the encapsulated enzyme was separated from the unbound CFE by ultracentrifugation (Discovery 90 SE, rotor T1270, Fisher Scientific, Loughborough, England) at 85,000× *g*, at 4 °C over 1 h as described by Nongonierma *et al*. [14]. PepX activity was quantified using H-Gly-Pro-pNA (Bachem) as described by Nongonierma *et al*. [14]. The enzyme activity PepX was measured in the supernatant (unbound enzymes) and in the liposomes which were disrupted using the following procedure: 200 μL of liposomes vesicles resuspended in 25 mM Tris-HCl buffer at pH 7.4 were mixed with 600 μL of a 2% (v/v) aqueous solution of phospholipase (Novozyme, Bagsvaerd, Denmark) and Triton X-100 (Fisons Scientific, Loughborough, England). The enzyme encapsulation was determined as per Nongonierma *et al*. [14].

#### 3.5.3. Determination of Enzyme Activities in the Cheese Samples

Curd and cheese extracts were prepared to measure various enzyme activities (PepX and LDH). An amount of 20 g of fresh curd or cheese was mixed with 40 mL of 0.05 M potassium phosphate buffer at pH 7.0 in a sterile stomacher bag. This was homogenized in a Stomacher (IUL, Barcelona, Spain) for 5 min or until homogenous and 10 mL was centrifuged at 4 °C for 10 min at 10,000× *g* (Sorvall 5U centrifuge, Unitech, Dublin, Ireland). Then the resultant supernatant (1 mL) was added to an eppendorf tube and re-centrifuged at 13,000× *g* for 5 min (Eppendorf 5417C, VWR International, Dublin, Ireland). The final supernatant was subsequently diluted as required and assayed for various enzyme activities. The whey samples were directly diluted in the adequate buffer used for the enzyme assay. All enzyme assays (PepX and LDH) were carried out in triplicate for each cheese trial on the three different days of manufacture.

### 3.6. Microbial Analyses

Total cell counts were estimated in the RSM after inoculation of 303, after growth, in the permeate and the retentate and after microfluidization and freeze-drying. Plating was carried out on an LM17 medium (Merck, Darmstadt, Germany) to determine the total cell count and samples were incubated at 30 °C during 72 h.

Microbiological analysis of cheese extracts (see Section 3.5.3.) were carried out in duplicate at each sampling point. Starters and non-starter lactic acid bacteria (NSLAB) were enumerated as described in Hickey *et al*. [34].

### 3.7. Physicochemical Analysis of the Cheese Samples

#### 3.7.1. Cheese Composition at Day 14

Cheese composition for pH, fat, protein, NaCl (salt) and moisture was determined at day 14 as described by Hickey *et al*. [34].

#### 3.7.2. Determination of Individual Free Amino Acids

Individual free amino acids (FAA) were determined on 24% trichloroacetic acid (TCA) filtrates prepared directly from the whey or from a pH 4.6 water soluble nitrogen fraction of the curd as per Kuchroo and Fox [35]. Analysis was carried out using a Jeol JLC-500/V Amino Acid Analyzer (Jeol Ltd, Herts, UK) fitted with a Jeol sodium high performance cation exchange column and the results were expressed as μg/g cheese or whey. All analyses were carried out in duplicate.

#### 3.7.3. Measurement of the Water Activity of the Cheese Samples

Water activity of the cheese samples was determined in triplicate during ripening. Water activity of ~5 g grated cheese was measured at room temperature (21 °C) with an AquaLab Series 3T (Labcell, Hampshire, UK).

### 3.8. Cryogenic Scanning Cryogenic-Scanning Electron Micrography

Cryogenic-scanning electron micrography (Cryo SEM-Zeiss Supra 40VP field emission, Carl Zeiss AG, Darmstadt, Germany) was used to visualize the liposomes in the cheese samples. Cryofixation of the samples was carried out in liquid nitrogen and Cryo SEM analysis was carried out as per Gee *et al*. [36].

### 3.9. Sensory Evaluation and Volatiles

The cheese samples were cut into 2 cm cubes and evaluated at 10 °C by a descriptive sensory panel (*n* = 10, 9 females, 1 male, ages 22–46 years) with more than 200 h experience with the descriptive analysis of Cheddar cheese flavor. Descriptive analysis of flavor used a 15 point universal intensity scale with the Spectrum™ method [37,38] and a previously established cheese flavor sensory language [39,40]. Each panelist evaluated cheeses from each treatment replication in triplicate.

Analysis of Cheddar cheese volatiles was conducted by headspace solid-phase microextraction (HS-SPME) as per Kang *et al*. [41]. Briefly, 5 g of grated cheese were placed in 20 mL SPME vials. An internal standard (10 mL of 81 ppm 2-methyl-3-heptanone in methanol) was added to each vial. Vials were equilibrated for 25 min at 40 °C. Headspace sampling was carried out using a CTC Analytics CombiPal Autosampler (CTC Analytics, Zwingen, Switzerland) with a single DVD/Carboxen/PDMS 1 cm fiber (Supelco, Bellefonte, PA, USA.). Each sample was analysed in triplicate. The SPME fiber was injected at 5.0 cm into an Agilent 6890 gas chromatograph with a 5973 MSD (Agilent Technologies Inc., Santa Clara, CA, USA) inlet fitted with a DB-5ms (30 m × 0.25 mm i.d. × 0.25 µm) column. Desorption time was 5 min and injector temperature was set at 250 °C in splitless mode. The initial oven temperature was 40 °C for 3 min, raised to 90 °C at 10 °C/min then to 200 °C at 5 °C/min, held for 10 min at 200 °C then raised to 250 °C at 20 °C/min and held for 5 min at 250 °C. Helium was used as a carrier gas at constant flow rate of 1 mL/min. Detection scanning (temperature of the source and MSD transfer line: 250 °C, and of the quadrupole: 150 °C) from 35–350 *m/z* was performed to identify compounds of interest. Volatile compounds were identified using mass spectra comparisons to the NIST 2005 mass spectral library. Relative intensities of each compound were compiled from total ion counts for each peak of interest and compared to the peak area of the internal standard.

### 3.10. Statistical Analyses

For the enzyme assays, microbiological cell counts and composition analyses, one way analysis of variance (ANOVA) was carried out with SPS (version 9, SPSS Inc., Chicago, IL, USA). A Student Newman-Keuls test was performed for mean multi comparison test at a significance level *p* < 0.05. For sensory analysis and volatile analysis, one-way ANOVA with Fishers least significant difference was used to determine treatment effects. Principal component analysis (PCA) of the correlation matrix was also applied to characterize differences in descriptive attributes between cheeses (SAS, version 9.2, Cary, NC, USA).

## 4. Conclusions

Inclusion of liposomes into Cheddar cheese had no adverse impact on cheese composition or microbiology. A CFE from *Lactococcus lactis* supsb. *lactis* 303 encapsulated in liposomes partitioned with the curd during Cheddar cheese production, and prevented excessive losses of CFE into the whey during production. No significant differences (*p* ≥ 0.05) in primary or secondary proteolysis or expressed PepX activity were evident between the cheeses during ripening. Even though flavor development as quantified by sensory and volatile attributes were more profound in the liposomal encapsulated CFE cheeses in comparison to the Control cheeses, no differences were evident to cheeses containing only empty liposomes at 112 days. As the cheeses containing CFE encapsulated in liposomes had significantly (*p* < 0.05) less losses of PepX activity to the whey at drainage in comparison to the cheeses with added free CFE, nor had any additional expressed PepX activity during ripening in comparison to the other cheeses, and as intact liposomes were apparent within the cheese up to 28 days of ripening, it appears that these liposomes did not degrade during ripening. Thus the lack of liposome degradation in combination with low encapsulation efficiencies resulted in an insufficient amount of additional enzyme activity being available within the cheese curd to significantly impact proteolysis and, thus, flavor development. The liposomes used were comprised mainly of Prolipo Duo which has a low zeta potential and high stability and even though this is thought to be more beneficial for encapsulation efficiencies, it appears to have the opposite impact in terms of enzyme release in cheese curd during ripening. This result highlights the potential of liposomes to minimize losses of encapsulated enzymes into the whey during cheese production, but also highlights the need to optimize the hydrophobicity, zeta potential, size and composition of the liposomes not only for encapsulation efficiency, but also for stability within the cheese curd in order to maximize their use as vectors for enzyme addition in cheese to augment ripening.
